# Multicentric Castleman Disease: The Hyaline Vascular Type Without HIV Association in a Young Male With Cutaneous Manifestation and Renal Impairment

**DOI:** 10.7759/cureus.30323

**Published:** 2022-10-15

**Authors:** Abed H Khan, Mohammad Ferdous Ur Rahaman, Sadia Rahman, Mizanur Rahman Khan, Chowdhury Adnan Sami

**Affiliations:** 1 Internal Medicine, Bangabandhu Sheikh Mujib Medical University, Dhaka, BGD

**Keywords:** hyaline vascular type, renal impairment, cutaneous manifestation, hiv negative, multicentric castleman disease

## Abstract

Castleman disease (CD), a heterogenous lymphoproliferative disorder resulting from immune dysregulation, is a very rare disease in clinical practice. The clinical spectrum of Castleman disease is wide and its treatment options are mostly based on case reports and case series. To date, two clinical and four histological types have been described. It has recently been successfully demonstrated that the pathogenesis of multicentric Castleman disease (MCD) has a direct association with human immunodeficiency virus (HIV) and human herpes virus 8 (HHV-8) infection which is why further studies are necessary. Here, we report an unusual case of MCD not associated with HIV and having a histological diagnosis of the hyaline vascular type that presented with acute renal impairment and subcutaneous abnormal lymphatic proliferation.

## Introduction

First described by Castle et al. in 1956 [1], Castleman disease (CD) is a rare heterogeneous lymphoproliferative disease known as angiofollicular lymph node hyperplasia or large lymph node hyperplasia. The manifestations include asymptomatic lymphadenopathy, solitary lymphadenopathy, widespread lymphadenopathy, and severe systemic symptoms.

Histologically, three patterns are described: hyaline vascular type (80% to 90%); plasma cell type (10% to 20%), and the mixed form [2]. The clinical manifestations are unicentric or localized and multicentric or generalized, and later presented with systemic manifestations. Localized illness has a more benign progression and can be further subdivided into hyaline vascular and plasma cell types [3]. Typically, the plasma cell variety of multicentric Castleman disease (MCD) is observed. However, hyaline vascular and mixed associations have also been seen. Without therapy, MCD carries a worse prognosis, with mortality probable owing to infection or cancer [3]. Recently, a more aggressive variant of MCD has been described, namely the plasmablastic variant [4].

Human immunodeficiency virus (HIV) infection, with or without human herpes viruses 8 (HHV-8)/Kaposi's sarcoma-associated herpesvirus (KSHV) co-infection, is associated with MCD. Most cases of HIV-associated CD are plasma cell or plasmablastic variations with more severe symptoms and a fast-advancing clinical history [5-11]

Although uncommon, renal involvement may complicate the secondary manifestation of MCD. Among the reported renal consequences are nephrotic syndrome, acute renal failure, interstitial nephritis, thrombotic microangiopathy, renal lymphoma, and renal amyloidosis [12]. Skin symptoms are also uncommon. However, this case is noteworthy due to the unusual presentation of MCD with hyaline vascular type, acute renal failure, and subcutaneous lymphatic growth.

## Case presentation

A 28-year-old male from Narayanganj, Bangladesh was referred to our hospital in February 2017 with a two-month history of high-grade fever, weakness, and weight loss, followed by generalized swelling. He experienced a weight loss of about 6 kgs in two months. The swelling appeared about one month after the onset of symptoms and was associated with decreased urine output. General examination showed facial puffiness, mild anemia, elevated blood pressure (150/90 mmHg), and bipedal pitting edema. Discrete rubbery, non-tender lymph nodes (mostly involving the inguinal region, the largest one measuring 3 cm × 4 cm) in cervical, axillary, inguinal, and epitrochlear regions were also noted. Urine examination showed moderate proteinuria but no hematuria. Systemic examination revealed hepatosplenomegaly (liver span being 22 cm and spleen being 12 cm from left costal margin) and moderate ascites (evidenced by shifting dullness). One striking finding was hyperpigmentation with dry ichthyosis of the skin which he acknowledged for the last four to five years. One firm, mobile subcutaneous (6 cm × 5 cm) swelling with a positive slip sign was also noticed in the upper medial aspect of the right thigh. He mentioned having similar swellings in the left forearm and right gluteal region, which were surgically excised in 2013 and 2014 respectively (the histopathology reports are unavailable). Initially, differential diagnoses were in line with lymphoma and disseminated tuberculosis (TB) keeping in mind the possibilities as well.

Laboratory evaluation showed a hemoglobin level of 9.1 g/dl and a white blood cell count of 8.5×10^9^/L with 68% neutrophil. The platelet count was 215×10^9^/L, and the erythrocyte sedimentation rate (ESR) was 120 mm during the first hour. Mean corpuscular volume (MCV), mean corpuscular hemoglobin (MCH), and mean corpuscular hemoglobin concentration (MCHC) were all within normal limits with normochromic normocytic red cells. Routine urine examination showed moderate proteinuria, red blood cell 4-6/HPF, and pus cell 2-4/HPF without a cast but the urine culture was sterile. The 24-hour urinary total protein was 0.74 gm.

Biochemical analysis revealed serum creatinine 1.65 mg/dl; serum potassium 6.1 mmol/L; total protein 57 gm/L with serum albumin 19 g/L, globulin 38 gm/L and serum cholesterol 160 mg/dL. The liver function test revealed alanine aminotransferase 29 IU/L; aspartate aminotransferase 34 IU/L; serum alkaline phosphatase 310 IU/L; serum bilirubin (total) 0.8 mg/dl. Serum protein electrophoresis revealed polyclonal hypergammaglobulinemia with hypoalbuminemia.

Chest radiograph revealed interstitial infiltrates without hilar lymphadenopathy. Ultrasound scanning of the whole abdomen revealed hepatosplenomegaly, echogenic kidneys (normal-sized), and moderate ascites. Mild pericardial effusion was detected in echocardiography. The ascitic fluid study revealed 4.3 g/dl of protein and 250 cells/cumm (20% polymorphs, 80% lymphocytes), and was negative for malignant cells. The ascitic fluid culture was sterile. Screening for hepatitis B, hepatitis C, syphilis, and HIV were negative. Mantoux test was 2 mm after 72 hours. The toxoplasma antibody was also negative. The endoscopy of the upper gastrointestinal (GI) tract was unremarkable.

**Figure 1:**
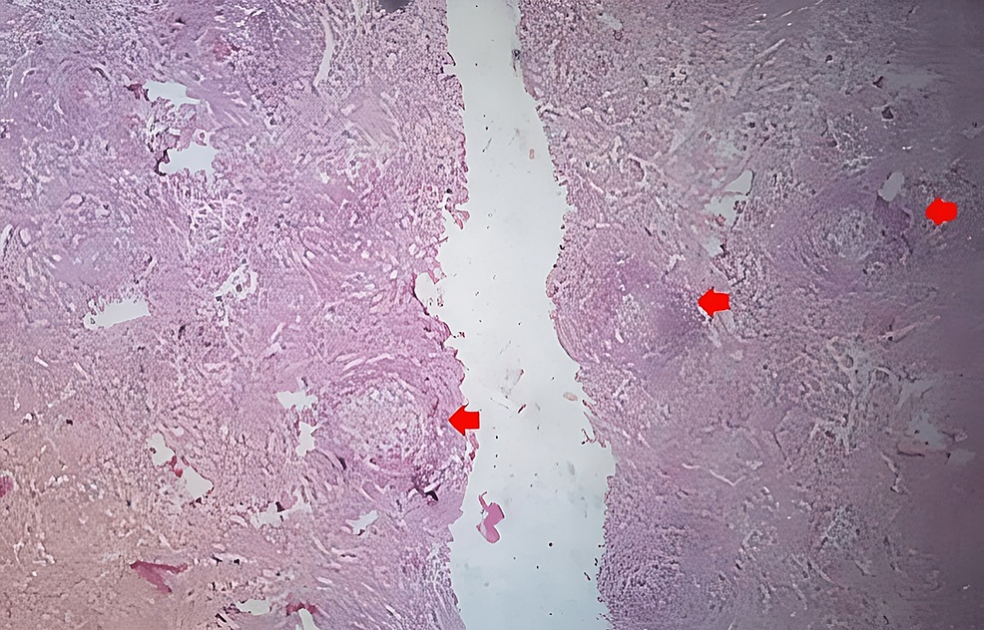
Histopathology from the cervical lymph node revealed hyperplastic lymphoid follicles having marked vascular proliferation and hyalinization.

**Figure 2:**
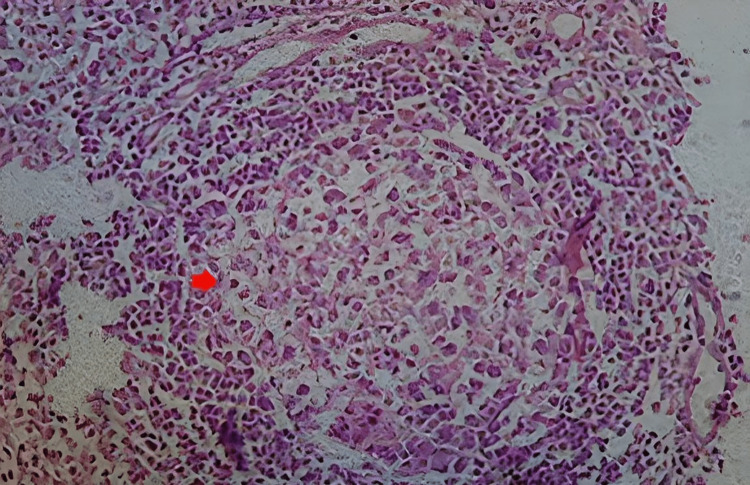
Histopathological examination of the cervical lymph node revealed hyperplastic lymphoid follicles with an atretic germinal center.

Histopathological examination from the cervical lymph node revealed hyperplastic lymphoid follicles interspersed with capillaries showing hyalinization, which was suggestive of hyaline vascular type CD (Figures 1, 2). Results of the duplex study and magnetic resonance imaging of subcutaneous swelling were suggestive of abnormal lymphatic proliferation.

Following initial management with injectable diuretic and albumin, the patient received oral prednisolone 50 mg and thalidomide 100 mg daily for 10 days. Although fever and peripheral edema subsided, ascites continued to progress. Then chemotherapy was started with rituximab, cyclophosphamide, etoposide, doxorubicin, and prednisolone. After two cycles of chemotherapy (each having a duration of 21 days), there was a significant improvement as evidenced by the absence of fever, marked regression of lymph node size, and disappearance of edema and ascites. His hemoglobin rose to 11.8 g/dl, and his erythrocyte sedimentation rate fell to 25 mm in the first hour. Kidney function tests were normal (serum creatinine 0.47 mg/dl; serum potassium 3.7 mmol/L). Proteinuria also subsided with 24-hour urinary protein 0.07 gm.

## Discussion

Castleman's disease was first described by Castleman et al. in 1954 when they described a patient having lymph node (mediastinal) enlargement with involution of germinal-center and marked proliferation of capillary with hyperplastic endothelium [1]. Four variants of CD have been described: hyaline vascular type, plasma cell type, mixed cellularity type, and plasmablastic variant. Clinically, the disease is described as either unicentric or multicentric CD. Multicentric Castleman disease usually presents with systemic involvement and carries a worse prognosis [2,3].

Chen, in 1984, reported the association of CD with Kaposi's sarcoma [5]. Oksenhendler et al. established the relationship between MCD and HIV [6]. Dupin et al. and others have demonstrated a direct association with HHV-8 [10]. Overproduction of interleukin (IL)-6 has already been recognized in the pathogenesis of CD by Yoshizaki et al. [11].

This was an unusual case as histological hyaline vascular type presented with features of MCD. The presentation was high-grade fever, fatigue, and significant weight loss followed by generalized swelling. Anemia, elevated blood pressure (BP), edema, generalized lymphadenopathy, hepatosplenomegaly, and ascites were key clinical findings. Investigations revealed low hemoglobin (Hb) with elevated ESR, proteinuria, active sediments in culture-proven sterile urine, elevated serum creatinine, hypoalbuminemia, and hypergammaglobulinemia. The histopathological examination of the lymph node established the diagnosis of the hyaline vascular type of CD.

Renal involvement is an uncommon secondary phenomenon. A study of 113 patients with CD showed only six among 113 patients had renal involvement [13]. In another study of 19 patients having CD with renal involvement, it has been shown that the presentations were proteinuria (95%), hematuria (78%), and acute renal failure (63%). Ours was a patient of MCD with proteinuria and acute renal impairment [14].

Skin involvement is very rare in CD. Cutaneous manifestations were mostly described as part of polyneuropathy, organomegaly, endocrinopathy, monoclonal paraproteinemia, skin changes, and sclerotic bone lesion (POEMS) syndrome [13]. The usual skin changes are hyperpigmentation, hyperkeratosis, finger clubbing, skin angioma, hemangioma, or a lymphangioma. Paraneoplastic pemphigus is another rare association. Our patient had hyperpigmented hyperkeratotic skin with subcutaneous abnormal lymphatic proliferation.

Currently, there is no consensus regarding the management of the disease. Surgical resection is often curative in unicentric CD not associated with HHV-8. The treatment options for MCD are steroids, chemotherapy (cyclophosphamide, doxorubicin, vincristine, and prednisolone (CHOP)), immunomodulators like thalidomide, interferon-alfa and monoclonal antibody. Treatment targeting IL-6 (anti-IL-6, suramin, and anti-IL-6 receptor antibody, atlizumab) can rapidly alleviate symptoms and induce disease regression with durable remission. Treatment with interferon-alfa, anti-CD 20 monoclonal antibody, and rituximab can also result in durable clinical and biochemical remission. Antiviral and highly active anti-retroviral therapy was indicated for HHV-8 and HIV-positive patients, respectively [15].

After initial treatment with oral prednisolone and thalidomide, our patient received chemotherapy (cyclophosphamide, doxorubicin, vincristine, and prednisolone along with rituximab (R-CHOP)). In total, six cycles were given. The patient’s final follow-up recorded a magnificent response with complete clinical and biochemical remission.

## Conclusions

Being a rare form of a lymphoproliferative disorder, CD is a clinical syndrome comprising fever with large lymph node hyperplasia along with plasma cell infiltration. Anemia, hypergammaglobulinemia, and an increase in the plasma level of acute phase reactants are also common along with known HIV and HHV-8 association. This case report is aimed at revisiting such an uncommon condition and emphasizing the necessity to keep in mind that not all forms of CD are associated with HIV or Kaposi's sarcoma. Histopathology is the key here to differentiate the disease entity from other variations of lymphoproliferative conditions. However, renal and cutaneous manifestation is to be sought out with its usual manifestation.
